# Genes’ Interactions: A Major Contributor to the Malignant Transformation of Endometriosis

**DOI:** 10.3390/ijms20081842

**Published:** 2019-04-14

**Authors:** Konstantinos Sapalidis, Nikolaos Machairiotis, Paul Zarogoulidis, Sofia Vasilakaki, Chrysanthi Sardeli, George Koimtzis, Efstathios Pavlidis, Athanasios Katsaounis, Dimitrios Giannakidis, Nikolaos Michalopoulos, Stylianos Mantalobas, Vyron Alexandrou, Charilaos Koulouris, Aikaterini Amaniti, Isaak Kesisoglou

**Affiliations:** 1 Department of Obstetrics-Gynaecology, Royal Oldham Hospital, Pennine Accute Trust, Oldham OL12JH, UK; sapalidiskonstantinos@gmail.com (K.S.); nikolaosmachairiotis@yahoo.com (N.M.); 2 3rd Department of Surgery, “AHEPA” University Hospital, Aristotle University of Thessaloniki, 55236 Thessaloniki, Greece; drgxkoimtzis@gmail.com (G.K.); pavlidis.md@gmail.com (E.P.); athanasios_katsaounis@hotmail.com (A.K.); giannakidis.d@gmail.com (D.G.); nmichalopoulos1@outlook.com (N.M.); steliosmantalobas@yahoo.gr (S.M.); vyrwnal@gmail.com (V.A.); charilaoskoulouris@gmail.com (C.K.); isaackesisoglou@outlook.com (I.K.); 3 Department of Chemistry, National and Kapodistrian University of Athens, Panepistimiopolis, 15771 Athens, Greece; svasilak@chem.uoa.gr; 4 Department of Pharmacology & Clinical Pharmacology, School of Medicine, Faculty of Health Sciences, Aristotle University of Thessaloniki, 52236 Thessaloniki, Greece; sardeli@auth.gr; 5 Anesthesiology Department, “AHEPA” University Hospital, Aristotle University of Thessaloniki, 52236 Thessaloniki, Greece; amanitik@gmail.com

**Keywords:** malignant transformation, endometriosis, cancer, tumor, mutation, biomarker

## Abstract

The genetic and epigenetic factors that contribute to the malignant transformation of endometriosis are still under investigation. The objective of the present study was to investigate the genetic link between endometriosis and cancer by examining and correlating the latest clinical observations with biological experimental data. We collected updated evidence about the genetic relationship between endometriosis and cancers by conducting a comprehensive search of PubMed and Scopus databases, focusing on the papers published between January 2018 and January 2019. New insights into the mechanism of the malignant transformation of endometriosis have been published recently. The use of state-of-the-art techniques and methods, such as the genome-wide association study analysis and the weighted gene co-expression analysis, have significantly altered our understanding of the association between endometriosis and endometriosis-associated cancer development. Interestingly, the interactions formed between genes seem to play a pivotal role in the phenotypic expression of mutations. Therefore, the effect of single nucleotide polymorphisms and the function of the expression quantitative trait loci on genes’ expression have been the subject of many recent works. In addition, it has been discovered that genes, the mutations of which have been related to the development of endometriosis, play a role as hub genes. This may lead to new areas of research for understanding the mechanism of malignant transformation of the disease. Significant steps forward have been made towards the identification of factors that control the malignant transformation of endometriosis. Still, due to rarity of the event, a better-organized scheme for sampling on a global level should be adopted.

## 1. Introduction

Although rare, malignant transformation of endometriosis is an active field of research. Many studies have focused on the identification of risk factors, which may be used for the early prognosis. Still, the results are poor due to the complexity of the event, and it is, therefore, of significant interest.

Endometriosis itself is a complicated disease. The fact that there is a variation of its genotype and phenotype has led to the development of several theories for its pathogenesis, but none of them may offer a certain explanation [1]. Therefore, the treatment of the disease heavily depends on the case and the history of the patient. Diagnosis of the disease is also a challenging task, despite the recent developments in this field [2].

Several different types of malignant tumors have been associated to endometriosis, mainly subtypes of endometriosis-associated ovarian cancer (EAOC) [3,4,5]. However, the continuously published case reports and cohort studies perplex the prediction of malignant transformation by revealing that the disease can progress to a variety of tumor types.

According to several cohort studies, the higher risk for the development of breast cancer and subtypes of skin cancer after the diagnosis of endometriosis has been underlined [6,7,8]. In addition, squamous cell carcinoma of the ovary, which is a very rare type of tumor, has been reported to be associated with or arising from endometriosis with an 80% patient mortality, within the first few months after diagnosis [9]. A case of clear cell carcinoma of the cervix has been also reported lately [10]. Ectopic endometrium was found attached to the tumor, which evidences the link between the two diseases.

Moreover, endometriosis-associated recto-sigmoid cancer was reported for a patient who had a surgery treatment of endometriotic ovarian cyst in her history [11]. Endometrial tissue was found in the intestinal wall and, although adjuvant chemotherapy was applied after the surgery treatment of the carcinoma, metastasis in the rectum was diagnosed. In a similar case, they reported the occurrence of a rectal lesion in a woman with a history of pelvic endometriosis and uterine myomatosis [12]. Based on the histopathology results, this lesion turned out to be adenocarcinoma arising from endometrial tissue in the colonic wall.

Another interesting case by Kilzieh et al. [13] describes the reoccurrence of endometriosis 13 years after the first surgery treatment. The histological study of the masses after their surgical resection revealed endometriosis—located at rectovaginal septum—and a low-grade endometrial stromal sarcoma arising from it. An older report by Tanas et al. [14] also manifests the possibility of a low progression of the disease. The report describes the case of a patient with endometriosis, which gradually transported to atypical endometriosis and endometrioid adenocarcinoma within a period of 10 years.

The development of different types of endometrial cells during the transportation of endometrium has been reported in other cases as well. For example, it has been reported the malignant transformation of endometriosis to neoplasia in the abdominal wall with an immunohistochemical profile of adenocarcinoma with clear cell components [15]. In this case, metastasis to an external iliac chain lymph node was also observed during lymphadenectomy.

Consequently, clinical reports demonstrate the need for tools to predict the disease’s progression. Recent studies have used epidemiological data to identify the principles of endometriosis’ transformation. In a recent large cohort study, the increased risk for the development of ovarian cancer (OC) to women with surgical diagnosis of endometriosis was confirmed [15]. It is worth mentioning here that cohort studies have shown that EAOCs have an early diagnosis and an improved rate of patients’ survival [16,17,18,19,20,21,22].

The study by Zhou et al. [23] performed a risk factor analysis for the prediction of the ovarian endometriosis transformation. They reported that the duration of endometriosis is a dependent factor of malignant transformation, an outcome that has also been produced by an older study using a larger sample. [24] Zhou et al. also linked the risk of transformation with an elevated body mass index value after menopause and the occurrence of thyroid disease. They also indicated that early age of onset, dysmenorrhea, irregular vaginal bleeding, number of abortions, myoma of uterus, and multiple foci of endometriosis may be used as risk factors for transformation. In addition, cases with advanced stage of endometriosis (stage III or IV) seem to possess a higher risk of OC appearance, [25] while the type of endometriosis has been related to the risk of neoplasia [26].

Genome analysis has also been used for the characterization of the genetic profiles of endometriosis and endometriosis-associated cancers (EAC) in an attempted to understand the molecular events which lead to transformation. Despite the limitations of the relevant analytical methods, recent developments, such as the whole-exome sequencing technique, have greatly advanced research in this field. However, both endometriosis and cancer are multifactorial disorders which are affected by multiple genes, clinical characteristics and environmental factors, while both change their molecular profiles as they progress.

In this review, we have collected recent original studies in order to present an update of genetic contributors and possible markers of endometriosis progression. We also mention the use of advanced analytical methods and techniques for genome analysis and their future potentials.

## 2. Methods

This mini literature review includes articles from January 2018 to January 2019, aiming to describe the latest works in the identification of genetic and epigenetic factors that contribute to the malignant transformation of endometriosis. The words “endometriosis” and “cancer” or “tumor” were used for searching PubMed and Scopus databases.

The inclusion criteria for accepting a paper were: (1) The investigation of genetic and epigenetic factors which link endometriosis to the occurrence of cancer, (2) The identification of genes’ alterations under the disease condition, (3) The clear and adequate demonstration of methods used to conduct the research. The exclusion criteria were: (1) The presentation of genetic/epigenetic factors that may be used for the treatment of endometriosis, (2) The presentation of clinical methods to monitor malignant transformation of endometriosis, (3) Any letters to the editor and editorials. Only English language articles were referred.

The initial search returned 314 papers in PubMed and 351 in Scopus (including duplications), and the titles, abstracts and methodologies were reviewed by the authors of this review. Finally, 37 papers (original papers, case studies, cohort studies, reviews) met the inclusion criteria, while one paper was excluded due to invalid methodology and contradictory conclusions.

## 3. Genome Profile of Endometriosis and Associated Cancer

In general, the process of acquiring genome data is challenging. This is due to the limitations arising from the methods used for the collection of the samples, the standard pathology laboratory tissue fixation techniques, and techniques for sequencing the samples. Despite this, significant progress has been made towards the understanding of biological events which lead to malignant transformation of endometriosis.

In general, recent studies have been performed towards the identification of mutated genes of endometriosis and EAC, and the investigation of targeted genes and proteins’ roles in the malignant transformation. 

### 3.1. Genetic Profile of Endometriosis and Endometriosis-Associated Cancers

Two studies tried to define differences between endometriotic and normal samples mutations’ profile. The first one is by Suda et al. [27], who performed a whole-exome sequencing on 13 endometriotic and 11 normal uterine endometrial epithelium samples, without concurrent gynecological cancers. Across all samples, mutations on cancer-associated genes (*KRAS*, *PIK3CA*, *FBXW7*, *PPP2R1A* and *PIK3R1*) were repeated, and they verified that the somatic mutations on both groups differed from the corresponding germline variants. This is one of the few studies which analyzes germline variants.

In addition, they proposed that the clonal expansion is led by epithelia cells with cancer-associated mutations in ectopic sites, due to their growth advantages. However, mutations on cancer-associated genes are not enough to promote malignant transformation of endometriosis. They also described the heterogeneity in mutation patterns of cells from different uterine origins of the same individual.

The second one is by Lv et al. [28], which tried to shed light on the pathogenesis of endometriosis by studying genes’ mutations in eutopic and paired eutopic endometrium samples. Their study included 30 patients with endometriosis in the absence of malignant or inflammatory diseases and a control group. Authors reported that they were unable to identify *BRAF* mutations in any sample, including the healthy ones. They also concluded that *CREB1* may act as transcription factor of *BRAF* activation. They assumed that their observations may show the role of *BRAF* in the promotion, but not in the development, of the disease.

In addition to the above studies, somatic driver mutations were assigned to tissue samples of 46 deep infiltrating endometriosis (DE) and 40 incisional endometriosis (IE) patients by Lac et al. [29], in an attempt to identify any shared profiles. Known somatic mutations in *KRAS*, *PIK3CA* and *ERBB2* were detected in a 10.0% of IE patients, while 22.2% of DE patients had *KRAS* or *CTNNB1* mutations. Authors noted that the hotspot mutations on *CTNNB1*, *PIK3CA* and *ERBB2* were only in the glandular-epithelial compartment of endometriotic lesions. They concluded that the rate of mutations and mutation profile between IE and DE are similar, and this may manifest the uterine as the origin of both.

Noticeably, they did not detect any somatic cancer-driver mutations in eutopic endometrium samples of four patients. Although the number of samples is relatively small, this finding may further support the idea that the driver mutations are promoted by the lesion’s formation, and they do not pre-occur. [30] Additionally, the loss of *ARID1A* protein was observed in a single case of DE, while loss of *PTEN* occurred in 18% of IE and 14% of DE patients. These observations were made only in the epithelial compartment of endometriotic lesions, and only in some generally clustered glands. This fact is in accordance with the clonal expansion and/or diffusion theory of endometriotic epithelial cells, originating from the endometriotic lesions, by the epithelial-mesenchymal transition process [30].

Several studies were carried out attempting to identify the effect of endometriosis on the genetic characteristics of EAC. Zhang et al. [31] investigated the molecular signatures of ovarian endometrioid adenocarcinoma (OEA) with and without concurrent endometriosis. They performed genome-wide transcriptome profiling analysis (20 mRNA samples), which revealed differences in the expression trend of 682 unique genes between the two groups. This affected 442 biological pathways in OEA with endometriosis, including NF-κB, RAS, TGF-β and inflammatory signaling pathways.

Kim et al. [32] compared the exome sequencing of serum samples and cancer tissues of five Korean patients with pathologically-diagnosed ovarian clear cell carcinoma (OCCC) arising from endometriosis, with 10 patients pathologically diagnosed with OCCC without endometriosis. The authors performed whole-exome sequencing and observed known and novel somatic mutations (Table 1). 

Although a common copy number variation (CNV) profile was not detected between the patients of the two groups, the 8q (47%) and 4q (80%) chromosomes were highly affected by arm-level gains and losses. CNVs were also detected in key pathways and oncogenic genes (Table 2). 

The authors concluded that similar genetic alterations occurred between the two groups of patients.

Additional to Kim et al. results, Shibuya et al. [33] ran a whole-exome sequencing on genomic DNAs from OCCC tissues and the corresponding non-cancerous tissues from 48 Japanese patients. They identified 53 somatic mutations related to *ARID1A*, *PIK3CA*, *KRAS* and *MSH6* genes. For patients with 23.6% C to G transition events, the Apolipoprotein B mRNA editing enzyme, catalytic polypeptide-like (APOBEC)-related mutations were more frequent, suggesting that APOBEC activation is associated with the carcinogenesis for these cases. 

The authors reported novel germline mismatch repair (MMR) mechanism gene alterations (*MSH2*: S900L, *MSH6*: M1326fs and R1095C) in three hypermutated cases, accompanied by other MMR somatic mutation in the tumor (*MLH3*: D813E, *PMS1*: V752I, *PMS2*: G105D), while the patients had no family history of malignancy. The role of these mutations in the pathogenesis of OCCC was highlighted.

Authors also reported mutations which related to chromatin remodeling and PI3K pathways, not previously related to OCCC, such as *PIK3R1* and *BUB1B*. They also observed that in the absence of mutations in *ARID1A* or oncogenic pathways mediated by the *PI3K* or *RAS* genes, mutations on other genes with the same effect on chromatin remodeling and PI3K pathways occurred. These novel mutations included *PDE4DIP*, *PDGFRB*, *ROS1*, *SMARCA4*, *NOTCH2*, *BRCA2*, *CDC73*, *ARID1B*, *MYH9*, *EBF1* genes. Finally, they reported that somatic mutations on *ZFHX4* gene were found in cases with aggressive OCCC, while mutations on *BRAF*, *ERBB2*, *PDGFRB*, *PGR*, and *KRAS* should guide the selection of chemotherapeutic agents.

An analysis on genome-wide association study (GWAS) datasets was performed by Painter et al. [34] for endometriosis and endometrial cancer. They discovered a genetic connection between the two diseases, formed by a nominally significant pleiotropy and an unexpected number of single nucleotide polymorphisms (SNPs) which were associated with the same direction of effect for both diseases. The SNP variation in the *SKAP1* region, which relates with OC and may function as expression quantitative trait loci (eQTLs) for *SKAP1* expression, as well as its neighboring genes (*HOXB2* and *HOXB3*), was highlighted.

An interesting study with outcomes relevant to epithelial-mesenchymal transition and its contribution to the development of malignancy was presented by Ponandai-Srinivasan et al. [35]. They isolated multipotent stem cells/progenitors from endometrium (18 samples) and endometrioma (11 samples) of endometriosis patients. They observed upregulation of *KIT*, *HIF2α* and *E-cadherin*, downregulation of *PTEN* and *ARID1A* and a disturbed ratio of *ER-β/ER-α* expression in four samples, which could be considered as a predisposition to gene mutations, effecting the phenotype of benign endometriotic multipotent stem cells toward malignancy. According to their hypothesis, different types of endometrium cells migrate to and are implanted in the ovaries during menstruation, and the local conditions of inflammation and hypoxia enhance DNA damaging. This gradually leads to the phenotypic transition of the stem cells and may result in the conversion of ovarian endometriosis to EAOC.

Finally, Fung et al. [36] investigated the effect of the menstrual cycle on gene expression by using a large number of endometrial samples (229 women of European ancestry). They confirmed fluctuations in the mean expression levels induced by the cycle stage, and that the oestrogen response, *KRAS* signaling up, and epithelial to mesenchymal transition are some of the affected pathways. The authors stated that, based on their observations, there is no radical difference in the mean expression levels of genes between endometriosis (eutopic endometrium) and control group.

Significantly, they discovered 45,923 *cis*-eQTLs, which associate with 417 unique genes, and additionally, another 2968 *trans*-eQTLs, which associate with 82 unique genes. They identified two of them in a region which relates with endometriosis risk and eQTLs, which seems to affect genes related to reproductive diseases. Of the aforementioned genes, 10% are transcription factors. 

To sum up, clonal expansion and epithelial to mesenchymal transition have been associated with the transformation mechanism in a number of recent studies, and therefore, are of great interest. Significant results have also been generated by the analysis of GWAS, eQTLs and SNPs, and more studies should be carried out on this base. 

### 3.2. Possible Contributors to and Biomarkers of the Malignant Transformation of Endometriosis

Several studies demonstrated their findings about the role of specific genes and proteins in malignant transformation of endometriosis in an attempt to present new markers for prognosis.

Zou et al. [37] focused on the identification of *CARD10* and *CARD11* gene mutations in ovarian endometriosis and they presented: a. A *CARD10* frame deletion (c.785_790delAGGAGA, p.K272_E273delKE) with a co-occurrence of uterine leiomyoma, b. A *CARD10* frame deletion (c.785_802delAGGAGAAGGAGAAGGAGA, p.K272_V277delKEPDNV) in a patient with a decreased blood eosinophil granulocyte ratio and an elevated serum creatine kinase level, c. A heterozygous missense mutation in *CARD11* (c.49G > T, p.D17Y) accompanied by an increased level of cancer antigen 125, and d. A heterozygous missense mutation in *CARD11* (c.160G > C, p.E54Q). The two mutations in *CARD11* demonstrated a high conservation among the 21 vertebrate species from GenBank.

Additionally, Guo et al. [38] worked on the identification of *CTCF* mutations in Chinese patients and identified two mutations in two different patients: the novel mutation p.K206E (c.616A > G) in a patient with primary infertility, and p.H373L (c.1118A > T) mutation in a patient diagnosed with uterine leiomyoma.

An interesting transformed endometriotic cell model was created by Rockfield et al. [39], characterized by the absence of cellular senescence and the dysregulation of mRNAs encoded by genes related to iron and epithelial-mesenchymal transition pathways. They intended to study the role of the intercellular levels of iron in the malignant transformation of endometriosis to specific subtypes of OCs by monitoring the expression of Nuclear Receptor Coactivator 4 (*NCOA4*). They observed an increased expression of both *NCOA4* isomers in the transformed endometriotic cells and an additional increased expression of NCOA4α protein in the cases of OC cells. The absence of *NCOA4* is characterized by a reduction in cell survival.

Yeo et al. [40] identified 14 types of C-type lectin receptors (CLRs) and their adaptor molecules in the intraperitoneal fluid and a differentiation in their pattern expression between benign diseases, endometriosis, and malignant tumors samples. Furthermore, they associated MR2, Dec-205 and Galectin mRNAs with the progression of endometriosis and its metastatic characteristics. Overall, authors reported the similarity of immunologic characteristics between endometriosis and gynecologic cancers.

Further work has been done by Chang et al. [41], who documented the crucial role of inflammation in the malignant transformation of endometriosis by analyzing microarray data. They observed that 7 genes of inflammasome complex (*Caspase-4*, *Caspase-7*, *Caspase-8*, *NLRP3*, *AIM2*, *PYCARD*, *NAIP*) and 11 genes of the inflammasome-related pathway (*IL-1B*, *IL1RL1*, *IL-18*, *TLR1*, *TLR7*, *TOLLIP*, *NFKBIA*, *TNF*, *TNFAIP3*, *INFGR2*, *P2RX7*) were highly expressed in endometriosis-associated ovarian carcinoma and demonstrated a tendency to correlate with poor survival.

Ciavattini et al. [42] studied the involvement of the unfolded protein response (UPR) in the transformation of endometriosis to endometrioid ovarian carcinoma. They confirmed the role of *ATF6* and *GRP78* in tumor development and the oncosuppressive role of *CHOP* and *XBP1*, the lack of which seems to promote the malignant transformation. They also suggested that *XBP1* could be used to monitor neoplastic transformation.

Another process that has been incriminated for the malignant progression of endometriosis is the oxidative stress, stimulated by the pathophysiology of the disease. Fujimoto et al. [43] proposed heme oxygenase-1 (HO-1) as a possible serum biomarker to monitor transformation and fluctuations of the 8-hydroxy-2-deoxy guanosine (8-OHdG), HO-1, total antioxidant capacity (TAC) and heme iron concentrations to be characteristic of each stage of tumor growth, mainly the initiation and progression stages. A twisted pattern in the tumor microenvironment formed by the upregulation of anti-oxidant TAC/heme iron and the lower levels of 8-OHdG and HO-1 concentration was proposed to promote malignancy.

Another factor that may contribute to the malignant transformation of endometriosis is noncoding RNAs (ncRNAs). Pan et al. [44] used endometrial cancer tissues and concluded that miR-302a-3p and miR-3130-3p were downregulated with an oncosuppressor role. In addition, the similar role of other miRNAs in endometrial cancer has been recently identified, opening a new possibility in the treatment and prognosis of endometrial cancer [45].

## 4. Discussion

Based on the above studies, we could signalize a few facts that need further consideration. Firstly, Suda et al. pointed out that the mutations between endometriotic and normal samples were similar to but different from the germline mutations, and with different mutant allele frequencies. Painter et al. also supported that endometriosis and endometrial cancer have common genetic predisposition factors, while underlining the significant role of SNPs. It was also showed the significance of the interaction between genes in the regulation of gene expression. These facts demonstrated the shared genetic background between different types of endometriosis, EAC and normal endometrium, despite their different phenotype.

In a recent review, Kammenga [46] analyzed the phenomenon of having different phenotypes corresponding to the same mutations, which is a challenging biological topic. The author underlined the fact that it is not the allelic variation of the mutated genes responsible for the different phenotypes, as it was thought, but rather the genetic background of these genes, defined as their interactions with other genes. Changes in this network of interactions may result in different levels of the phenotypic expression of mutated genes. Moreover, the role of cryptic genetic variants was also mentioned, a single-nucleotide variant type which have a phenotypic effect only under atypical conditions and environmental factors in these interactions, and so in the expression of mutated genes. 

In the same concept, the work of Castel et al. [47] demonstrated the effect regulatory variants (eQTLs) may have on coding variants (nsSNPs), which results in the alteration of these genes’ expression (measured as the amount of protein or RNA they produced), and consequently, the phenotype. They used cohorts of cancer and autism cases to evidence that regulation of coding variants with a “disease cause” phenotype is contingent on individual’s eQTL and the genotypes of coding variant. 

The role of eQTLs and SNPs has been investigated in endometriosis, with some interesting outcomes. As mentioned above, the work of Fung et al., which mapped eQTL in endometrial tissue samples, confirmed the association between endometrial eQTL expression levels and risk SNPs in rheumatoid arthritis and inflammatory bowel disease, among others. Interestingly, a recent case report [48] described the rare case of a patient with endometriosis and a long history of non-gynecological comorbidities, including autoimmune diseases such multiple sclerosis and Crohn’s disease. This case seems to confirm the findings of Fung et al. The effect of SNPs on the progression of and development of endometriosis was also confirmed by a recent study [49] showing that these events control the disease.

Similarly to Fung et al., the work of Bakhtiarizadeh et al. [50] used weighted gene co-expression analysis [51] to investigate changes in gene co-expression network among the different stages of endometriosis. For their analysis, they used minimal to mild, mild to severe endometriosis and control group samples. Based on their findings, authors suggested that the alterations in the connectivity among genes may be the cause for endometriosis development, rather than the genes’ expression profiles, which apparently were similar between pathological and normal conditions. Hypothetically, this might be the case for the progression from benign to malignant, and therefore, such analysis would be highly desirable. Authors also identified and monitored the role of hub genes. [52] In short, the function of hub genes is to minimize the phenotypic effect of mutated genes. Interestingly, their role was not conserved among the different stages. Some known genes were identified as hub genes, including *MMP-2*, *PTEN*, *KRAS*, *HOXB6* and progesterone receptors.

In the light of the above studies, we could introduce a hypothesis here, in that different types of endometriosis and EACs—if not other type of cancers as well [53]—are the different phenotypes of the same “disease cause” genes. We could assume that cases in which severe endometriosis appears [54], or severe EAC occurs without synchronous endometriosis [55], are the phenotype of homozygous carriers. At the same time, we cou6d suggest that the cases which are characterized by lack of severity and the development of both endometriosis and EAC are the phenotype of heterozygous carriers. An interesting case of EAC which may fall into this category is endometrioid borderline ovarian tumors (EBOT), a rare disease, and therefore insufficiently studied. In a recent cohort study by Jia et al. [56], it was observed that women developed endometrial disorders (e.g. endometrial cancer, endometrial hyperplasia with or without atypia) after the initial EBOT surgery, and that no deaths occur due to the disease.

However, several studies support the idea that contributors to malignant transformation of endometriosis are epigenetic factors [57], that are developed under the unique microenvironment induced by the disease, such as the pressure for fibrogenesis [30]. Therefore, taking into account both theories and clinical observations, we could assume that there are two conditions under which the disease’s progression occurs. In the first one, the progression of endometriosis is dependent on epigenetic effects and, in this case, malignancy is an expected step in a series of sequential molecular events, and in the second one, it is controlled by hereditary mutated genes. It might well be a combination of these two, depending on the case. For example, it is well-defined that the local environment in the ovary promotes tumor growth. Thus, EAOC development may be driven mainly by epigenetic events. In addition, the cases which are characterized by a very slow progression, and finally, transformation of endometriosis [58,59] may also be an example of transformation due to epigenetic factors. Certainly, more data are needed for the genetic background of endometriosis and EAC and the related somatic and germline mutations in order to further support this hypothesis.

## 5. Conclusions

Key biological pathways, genes’ expression and regulation of proteins are subjected to alteration during the menstrual cycle. Additional, progression of endometriosis—from mild to severe to atypical to malignant—also induces relative changes. Therefore, identifying the factors that contribute to malignant transformation of endometriosis is a challenging task. Due to rarity of the event, adequate sampling has not been achieved yet. A better organized scheme for sampling on a global level should be forwarded, possibly by raising the awareness of clinicians, establishing a protocol for recording/sampling/testing in order to have well-documented and comparable results/observations, and creating a web database to collect the findings. 

## Figures and Tables

**Table 1:** Somatic mutations in 15 Korean OCCC patients.

Novel Mutations	Known Mutations
*ARID1A* (pLeu2011Gln, pAsn115His, pLeu1309Phe)	*PIK3CA*, *TP53*, *ARID1A*, *ARID2*, *KRAS*, *LRP1B*, *PPP2R1A*, *PTEN*, *SYNE1*, *ERBB2*, *RFX3*, *MED12*, *GPC3*, *MST1R*
*ERBB2* (pGly344Cys, pPhe976Leu)	
*GPC3* (pPro273His)	
*LRP1B* (pSer2444Pro, pLeu38Phe)	
*MED12* (pLeu1489Val)	
*MST1R* (pAla234Thr)	
*PTEN* (pArg335Gln)	
*RFX3* (pAla488Thr, pGln97Glu)	
*SYNE1* (pAsn5174Ile, pArg8641Trp, pAsn3630Thr, pPhe3401Leu, pAsp3294Ala, pThr112Ala)	

**Table 2:** Somatic copy number variations in key pathways and oncogenic genes in 15 Korean OCCC patients.

Key Pathways Mutated	Focal Level Gains of Oncogenic Genes	Focal Level Losses of Oncogenic Genes
TP53, PI3K/AKT, MAPK	*NTRK1*, (33%, 1q chromosome); *MYC*, (40%, 8q chromosome); *GNAS*, (47%, 20q chromosome)	*TET2*, (73%, 4 chromosome); *TSC1*, (67%, 9q chromosome); *BRCA2*, (60%, 13q chromosome); *SMAD4*, (47%, 18q chromosome)
